# Transcriptome and microbiota analysis reveal differences in the cecum of weaning pigs in response to different dietary crude protein levels

**DOI:** 10.5713/ab.25.0135

**Published:** 2025-08-12

**Authors:** Yu-Hsiang Yu, Sheng-Bing Chen, Han-Tsung Wang, Chuan-Shun Lin, Andrzej Dybus, Beata Hukowska-Szematowicz, Yi-Hung Li

**Affiliations:** 1 Department of Biotechnology and Animal Science, National Ilan University, Yilan, Taiwan; 2 Department of Animal Science and Technology, National Taiwan University, Taipei, Taiwan; 3 Agricultural Facilities and Environment Research Center, Agricultural Technology Research Institute, Miaoli, Taiwan; 4 Department of Genetics, West Pomeranian University of Technology in Szczecin, Szczecin, Poland; 5 Institute of Biology, University of Szczecin, Szczecin, Poland; 6 Molecular Biology and Biotechnology Center, University of Szczecin, Szczecin, Poland

**Keywords:** Crude Protein, Growth, Microbiota, Transcriptome, Weaning Pig

## Abstract

**Objective:**

This study explored the effects of different dietary crude protein (CP) levels on the cecal transcriptome and microbial composition in weaning pigs.

**Methods:**

Ninety-six weaning pigs were randomly assigned into three groups designated as H, M, and L groups. The H, M, and L groups were administered 20% and 18%, 18% and 16%, and 16% and 14% CP during the early (1–14 days) and late phases (15–28 days), respectively.

**Results:**

The final body weight and average daily gain in the L group were significantly lower than those in the other groups (p = 0.008). The feed conversion ratio was lower in the H and M groups than in the L group (p = 0.01). Cecal transcriptome analysis revealed that heatmap and principal component analysis of differentially expressed genes indicated the presence of distinct clusters among the groups. Genes associated with cell proliferation and differentiation and inflammation were down-regulated in the M and L groups, compared with corresponding genes in the H group (p<0.001). Pathway enrichment analysis suggested that genes related to IL-17 signaling pathway was down-regulated in the M and L groups (p<0.05). Beta diversity analysis and heatmap for microbial composition and function indicated the presence of distinct clusters among the groups. Carbohydrate-fermenting bacteria, such as *Megasphaera elsdenii* DSM 20460 and *Blautia luti* DSM 14534, exhibited higher levels in the M and L groups compared with the H group (p≤0.05). The abundance of *Lactobacillus amylovorus* DSM 20531 was significantly greater in the M group than in the other groups (p≤0.05). The abundance of *L. amylovorus* DSM 20531 was positively correlated with growth performance. Integrated multi-omics analysis suggested significant similarities between the cecal transcriptome and microbiota (p<0.01).

**Conclusion:**

Reducing CP levels modulates cell growth and alleviates inflammation in the cecum. A low CP diet causes cecal microbiota composition shift and promotes the proliferation of carbohydrate-fermenting bacteria. Overall, 18% CP in an early phase and 16% CP in a late phase can substantially improve growth and gut health in weaning pigs.

## INTRODUCTION

Animals fed a diet rich in crude protein (CP) excrete excess nitrogen in feces and urine; this indicates a reduction in their nitrogen utilization efficiency. Digestive enzymes responsible for the digestion of energy and protein in weaning pigs exhibit low levels of activity, suggesting the inadequate digestion of excessive dietary CP contents [1]. Reducing the level of CP while ensuring adequate supplementation of essential amino acids in the diet of weaning pigs may help reduce production costs and environmental burden. Therefore, CP levels must be optimized in the diet of weaning pigs.

Reducing the level of dietary CP from 22.5% to 17% while supplementing amino acids can limit diarrhea incidence (DI) in weaning pigs without compromising production [2–5]. However, reducing this level from 20% to 16% compromises growth performance [6–8]. Average daily gain (ADG) and feed conversion ratio (FCR) were lower in weaning pigs fed a low-CP diet (16.6% and 14.6% CP in the early and late phases, respectively) than in those fed a high-CP diet (18.5% and 16.5% CP in the early and late phases, respectively) [9]. By contrast, reducing the level of dietary CP from 22% to 19% exerted no effect on the growth performance of weaning pigs [10].

The cecum can promote the conversion of indigestible fibers into energy-rich compounds through microbial fermentation and regulation of electrolyte and water absorption [11]. Previous study has reported that the number of bacterial population in the cecal digesta is higher than in the colonic digesta in pigs [12]. The amount of microbial metabolites in the cecal digesta is abundant compared with the colonic digesta of pigs fed diets containing high resistant starch [13]. Excessive undigested protein that enters the cecum through the ileo–cecal junction undergoes microbial fermentation, leading to the proliferation of pathogens and the production of toxic substances [14,15]. These toxic substances can induce local and systemic inflammation, compromise intestinal epithelial integrity, and impair gut function, thereby causing postweaning diarrhea [4,14,15]. Reducing CP levels in pig diets mitigates the risk of postweaning diarrhea [16]. High CP levels disrupt the expression of water channel genes in the ileum, causing imbalances in the absorption and secretion of water and electrolytes [17]. Few studies have focused on the effects of different dietary CP levels on the intestinal transcriptome and gut microbiota of weaning pigs. Moreover, a systematic analysis (through high-throughput sequencing) of cecal gene expression and microbial composition in weaning pigs fed diets containing different CP levels remains to be conducted.

Interactions between the host, gut microbiota, and diet in nutrient digestion and absorption influence the growth of pigs. In this study, we hypothesized that reducing the level of dietary CP would change the intestinal gene expression and gut microbial composition, thereby influencing growth performance in weaning pigs. Specifically, we analyzed changes in the growth performance, cecal transcriptome and cecal microbial composition of weaning pigs in response to diets containing different CP levels.

## MATERIALS AND METHODS

### Animals and diets

All experiments were performed in accordance with established guidelines. The animal protocol was approved by the Institutional Animal Care and Use Committee of National Ilan University, Taiwan (reference: 113-5). A total of 96 weaning pigs ([Landrace×Yorkshire]×Duroc), with an average body weight (BW) of 7.6±0.03 kg, were fed diets containing different CP levels for 28 days. The experimental period was divided into early (1–14 days) and late (15–28) phases. On the basis of average BW, the pigs were randomly divided into 3 diet groups (4 pen replicates with 8 pigs [4 male and 4 female pigs] per pen) called the H, M, and L groups. The H group received 20% CP in the early phase and 18% CP in the late phase. The M group received 18% CP in the early phase and 16% CP in the late phase. Finally, the L group received 16% CP in the early phase and 14% CP in the late phase. Compositions of the 3 experimental diets are presented in Table 1. All diets were supplied in the form of meals and formulated to meet or exceed the requirements of pigs, as outlined by the National Research Council [18]. The dietary levels of CP were analyzed after the diets were subjected to blending [19]. All pigs were allowed to consume feed and water *ad libitum*. Each pen was equipped with a single-sided feeder and a nipple drinker. During the experimental period, the BW of each pig was measured on days 1, 7, 14, 21, and 28 to determine their ADG. Feed consumption per pen were measured daily to calculate average daily feed intake (ADFI). FCR was calculated using ADFI and ADG. Mortality was monitored daily.

### Analysis of fecal score and diarrhea incidence

All pigs with consistent feces and diarrhea were monitored daily. Fecal consistency, which indicated the severity of diarrhea, was evaluated and scored as follows: 0, solid; 1, semisolid; 2, semiliquid; and 3, liquid. Scores of 0 and 1 were regarded as normal, whereas scores of 2 and 3 indicated diarrhea. DI (%) was calculated using the following formula: (number of pigs having diarrhea in each pen × diarrhea days/[total pigs in each pen × 28 days]) × 100. The fecal score (FS) and DI were estimated as described previously [20].

### Sample collection

Two pigs with BW closest to the average BW of pigs in each pen at the end of the experiment (day 28) were euthanized through electrical stunning followed by exsanguination. Blood samples were collected from the anterior vena cava of these pigs after euthanasia. All blood samples were collected in serum tubes for blood biochemistry analysis. Cecum tissues were collected for transcriptome analyses. Cecal digesta was collected (from the same pigs used for blood collection), frozen in liquid nitrogen, and stored at −80°C for short-chain fatty acid (SCFA) profiling and full-length 16S ribosomal RNA sequencing.

### Blood biochemistry

The blood samples were centrifuged at 1,600×g for 15 min at 4°C. After centrifugation, serum was carefully transferred to 1.5-mL plastic tubes and stored at −20°C until analysis. Serum levels of glucose, triglycerides, cholesterol, high-density lipoprotein, and low-density lipoprotein were measured using a Hitachi 7170 Chemistry Analyzer. In addition, serum levels of aspartate transaminase (AST), alanine transaminase, alkaline phosphatase, total protein, albumin, globulin, blood urea nitrogen (BUN), and creatinine were measured using ADVIA Chemistry XPT (Siemens Healthineers). Cortisol levels were measured using a commercial enzyme-linked immunosorbent assay kit (Thermo Fisher Scientific) per the manufacturer’s instructions.

### Small intestinal morphology analysis

The intestinal tissue samples were fixed in buffered formalin, dehydrated in ethanol, embedded in paraffin, and sectioned using a rotary sectioning machine (Rotary Microtome Microm HM 340E; Thermo Fisher Scientific). Three cross-sections were prepared for each intestinal tissue sample and stained with hematoxylin–eosin. A total of 10 intact and well-oriented crypt–villus units were selected for each intestinal cross-section. Villus height and crypt depth were measured per section by using an image processing and analysis system (Olympus), and the ratio of villus height to crypt depth was calculated.

### Short-chain fatty acid profiling

Cecal digesta (50 mg) was added to 1 mL of 10% isobutanol. After homogenization and centrifugation, the supernatant was mixed with 20 mM sodium hydroxide and chloroform. Then, the mixture was vortexed and centrifuged. The aqueous phase was mixed with isobutanol, pyridine, and isobutyl chloroformate and subjected to vortex and sonication. Next, hexane was added to the mixture, and the mixture was centrifuged. The supernatant was used for SCFA profiling, which was performed through gas chromatography–mass spectrometry (Bruker Scion 436 GC-MS System equipped with an Agilent VF-5ms capillary column). SCFA concentrations were calculated on the basis of standard curves and the peak areas of each SCFA.

### Transcriptome analysis

Total RNA was extracted from the cecum by using a TRIzol Plus RNA Purification Kit (Thermo Fisher Scientific) per the manufacturer’s protocol. RNA quality and yield were assessed using a Nanodrop 2000 spectrophotometer (Thermo Fisher Scientific). The RNA integrity number was determined using an Agilent Bioanalyzer 2100 (Agilent Technologies). Complementary DNA libraries were constructed using a TruSeq Stranded mRNA kit (Illumina) per the manufacturer’s instructions. The libraries were subjected to 2×150-bp paired-end sequencing on a HiSeq 2500 platform (Illumina). From the raw data, clean reads were obtained by trimming reads containing adapters and low-quality sequences by using Trimmomatic (ver. 0.38). Q20, Q30, and GC content of the clean data were calculated. The reads were mapped to the pig reference genome (Ensembl; database ver. Sscrofa 11.1) by using HISAT2 (ver. 2.1.0). From the RNA-Seq data, a table of fragments per kilobase of transcript per million mapped reads was created using Cufflinks (ver. 2.2.1). Differential gene expression was analyzed using edgeR (ver. 3.28.1) and DESeq2 (ver. 1.26.0). Heatmaps for differentially expressed genes and principal component analysis (PCA) were performed using the expression data for each gene from each sample. Gene ontology (GO) and Kyoto Encyclopedia of Genes and Genomes (KEGG) pathway enrichment analyses of the differentially expressed genes were performed using the R package clusterProfiler (ver. 3.14.3). Integrated multi-omics analysis (Procrustes analysis) of PCA, principal coordinate analysis (PCoA), and nonmetric multidimensional scaling (NMDS) was performed using the R package procGPA (ver. 1.2.7).

### Full-length 16S ribosomal RNA sequencing

Microbial DNA was extracted from cecal digesta by using the ZymoBIOMICS DNA Miniprep Kit (Zymo Research) per the manufacturer’s instructions. The concentration and purity of the microbial DNA were determined using a NanoDrop ND1000 Spectrophotometer (Thermo Fisher Scientific). Bacterial V1–V9 hypervariable regions of the 16S rRNA gene were amplified using forward 27F (5′-AGRGTTYGATYM TGGCTCAG-3′) and reverse 1492R (5′-RGYTACCTTGT TACGACTT-3′) barcoded primers. DNA libraries were constructed using the commercial SMRTbell library method (Pacific Biosciences) and purified using AMPure PB Beads (Pacific Biosciences). Full lengths of 16S rRNA genes were sequenced on a PacBio Sequel II sequencing platform (Pacific Biosciences). After sequencing, the raw reads were demultiplexed and filtered to obtain circular consensus sequencing reads. Denoising was performed using the DADA2 package (ver. 1.20) to obtain initial amplicon sequence variants. Chimeric sequences were removed using the UCHIME algorithm (ver. 8.1), thereby obtaining clean reads. Bacterial sequences with ≥97% sequence similarity were clustered into the same operational taxonomic unit by using USEARCH software (ver. 10.0). Operational taxonomic units were classified and annotated using the QIIME2 feature-classifier classify-consensus-vsearch (ver. 2022.11) and the GreenGenes2 reference database (ver. 2022.10). Alpha diversity values (species richness [Chao1 and Fisher alpha] and evenness [Shannon Index and Simpson Index]) were calculated using the MicrobiomeAnalyst online platform. To analyze beta diversity, PCoA and NMDS were performed using MicrobiomeAnalyst. Microbial function was predicted using Tax4Fun (ver. 1.1.5). Heatmaps and color correlations were plotted using the R packages pheatmap (ver. 3.6.3) and corrplot (ver. 0.84), respectively.

### Statistical analysis

Each pen was defined as an experimental unit. Normality for all variables were tested using the Shapiro–Wilk test in SAS (ver. 9.4, 2012; SAS Institute). The experimental groups were compared using one-way analysis of variance followed by Tukey’s honestly significant difference test. The Kruskal–Wallis test and Dunn’s pairwise test were used to determine significant between-group differences in microbial composition in the cecum. Statistical significance was set at p≤0.05. For transcriptome analysis, the false discovery rate method and Bonferroni correction were used to identify differentially transcribed genes and enriched functions. Genes with a p value of <0.05 and a |log2 (fold change)| value>0.5 were regarded as differentially expressed genes.

## RESULTS

### Effects of different dietary crude protein levels on the growth performance, blood biochemistry, and gut morphology of weaning pigs

The effects of different dietary CP levels on the growth performance of weaning pigs are summarized in Table 2. The results revealed that the BW at the end of the experiment was greater in the H and M groups than in the L group (p = 0.008). The ADG of weaning pigs from days 15 to 28 and days 1 to 28 was greater in the M group than in the L group (p = 0.014 and p = 0.008, respectively). FCR from days 1 to 14 was lower in the H group than in the L group (p = 0.046). FCR from days 1 to 28 was lower in the H and M groups than in the L group (p = 0.01). No significant between-group differences were noted in mortality during the experimental period. However, no significant between-group difference was noted in FS and DI during the experimental period. The effects of different dietary CP levels on the blood biochemistry of weaning pigs are summarized in Table 3. The levels of AST, BUN, and cortisol were lower in the L group than in the H group (p = 0.02, p = 0.032, and p = 0.007, respectively). The effects of different dietary CP levels on the gut morphology of weaning pigs are summarized in Table 4. The average height of the villi in the duodenum and jejunum was greater in the M group than in the L group (p = 0.004 and p = 0.003, respectively). The ratio of villus height to crypt depth in the duodenum and ileum of weaning pigs was greater in the H and M groups than in the L group (p<0.001 and p = 0.003, respectively). No significant between-group differences were observed in the cecal levels of SCFAs (Supplement 1).

### Effects of different dietary crude protein levels on the cecal transcriptome of weaning pigs

**Figure 1:**
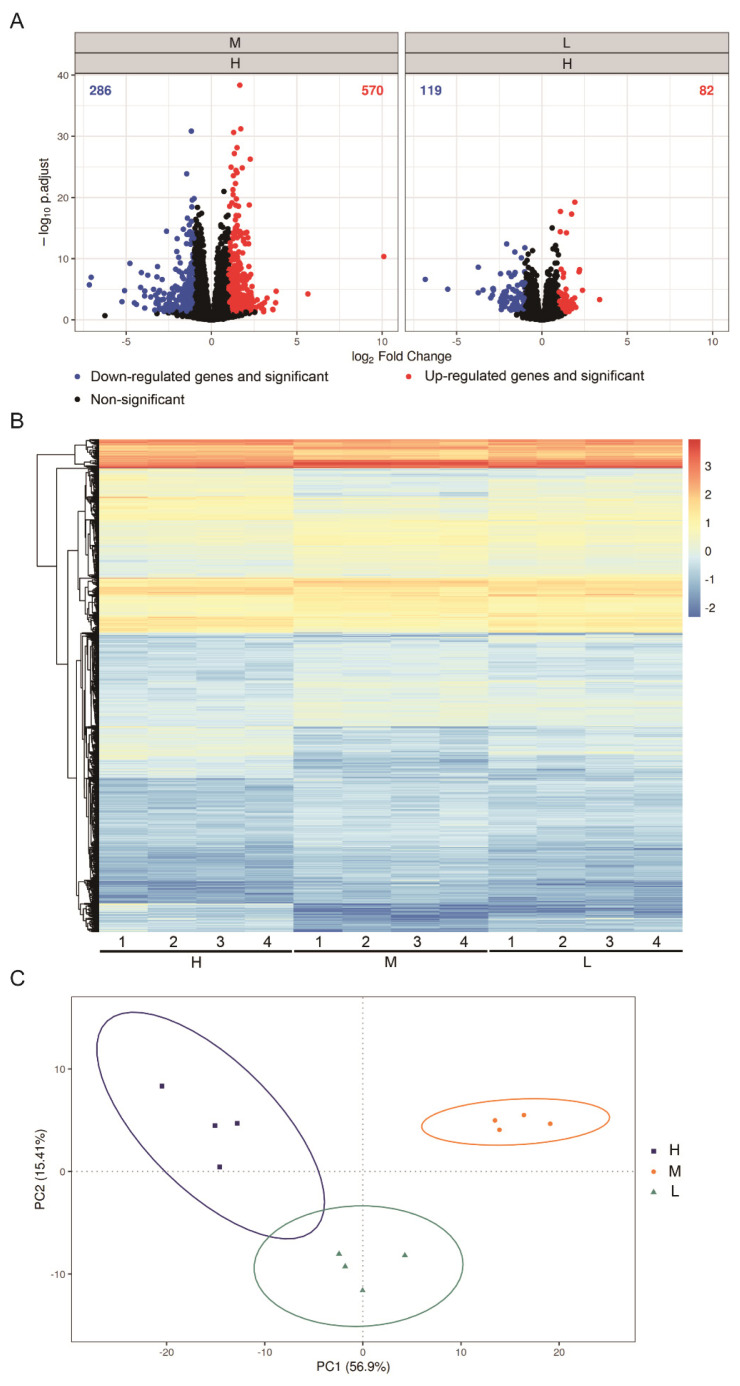
Gene expression in the cecum of weaning pigs fed diets containing different CP levels. (A) Volcano plot for differentially expressed genes in the cecum. The x-axis indicates the fold change in gene expression, whereas the y-axis indicates the statistical significance of the difference. (B) Heatmap for differentially expressed genes. Red and green indicate values greater and lower than the mean (average Z score) value, respectively. (C) Principal component analysis plot showing intra- and intergroup variations. H: supplementation of 20% CP in the early phase (1–14 days) and 18% CP in the late phase (15–28 days); M: supplementation of 18% CP in the early phase and 16% CP in the late phase; L: supplementation of 16% CP in the early phase and 14% CP in the late phase. Data were analyzed from 4 replicate pens per treatment. CP, crude protein.

A volcano plot depicting the general distribution of differentially expressed genes in the cecum of weaning pigs at the end of the experiment is presented in Figure 1A. Among the 856 differentially expressed genes, 570 were upregulated and 286 were downregulated in the M group compared with their expression levels in the H group. In the L group, 201 genes were differentially expressed compared with their expression levels in the H group. Of these genes, 82 were upregulated and 119 were downregulated. A heatmap analysis indicated that the supplementation of different dietary CP levels altered the transcriptome in the cecum of weaning pigs (Figure 1B). PCA revealed considerable between-group differences in this regard, with 56.9% and 15.41% of the variation explained by principal components PC1 and PC2, respectively (Figure 1C).

The effects of different dietary CP levels on the most regulated cecal genes at the end of the experiment are summarized in Table 5. The top 10 genes upregulated in the M group compared with their levels in the H group were phospholipase A2 group IIA (PLA2G2A; p<0.001), ankyrin repeat domain 34A (ANKRD34A; p = 0.002), docking protein 5 (DOK5; p = 0.019), chromosome 3 open reading frame 85 (C3orf85; p<0.001), NIMA related kinase 10 (NEK10; p = 0.046), and 5 unknown genes (p≤0.05). The top 10 genes downregulated in the M group compared with their levels in the H group were visinin like 1 (VSNL1; p<0.001), homeobox D13 (HOXD13; p<0.001), uridine phosphorylase 2 (p = 0.001), myocilin (p<0.001), secretoglobin family 1A member 1 (SCGB1A1; p = 0.002), potassium channel tetramerization domain containing 16 (KCTD16; p = 0.003), solute carrier family 5 member 8 (SLC5A8; p<0.001), carbonic anhydrase 7 (CA7; p<0.001), and two unknown gene (p<0.001). The top 10 genes upregulated in the L group compared with their levels in the H group were chromosome 3 open reading frame 85 (C3orf85; p< 0.001), sucrase–isomaltase 34A (SI; p = 0.009), collagen type XI alpha 2 chain (COL11A2; p = 0.004), cytochrome P450 family 4 subfamily F member 22 (CYP4F22; p<0.001), and 6 unknown genes (p<0.01). The top 10 genes downregulated in the L group compared with their levels in the H group were HOXD13 (p<0.001), VSNL1 (p<0.001), secretory leukocyte peptidase inhibitor (p<0.001), carbonic anhydrase 7 (p<0.001), potassium-transporting ATPase subunit beta (ATP4B; p<0.001), betaGal beta-1,3-N-acetylglucosaminyltransferase 7 (B3GNT7; p<0.001), lipopolysaccharide-binding protein (LBP; p<0.001), WAP four-disulfide core domain 5 (WFDC5; p<0.001), myocilin (MYOC; p<0.001), and one unknown gene (p<0.001).

The effects of different dietary CP levels on the cecal KEGG pathway of weaning pigs at the end of the experiment are summarized in Table 6; downregulation or upregulation was determined relative to the H group. The most upregulated KEGG pathways in the M group were cytoskeleton in muscle cells (p<0.001), cAMP signaling pathway (p<0.001), neuroactive ligand-receptor interaction (p<0.001), calcium signaling pathway (p<0.001), adrenergic signaling in cardiomyocytes (p<0.001), motor proteins (p<0.001), vascular smooth muscle contraction (p<0.001), cardiac muscle contraction (p<0.001), axon guidance (p = 0.002), nicotine addiction (p = 0.006), dilated cardiomyopathy (p = 0.016), glutamatergic synapse (p = 0.025), insulin secretion (p = 0.025), circadian entrainment (p = 0.047), and hypertrophic cardiomyopathy (p = 0.047). The most downregulated KEGG pathways in the M group were the IL-17 signaling pathway (p = 0.027), cytosolic DNA-sensing pathway (p = 0.043), TNF signaling pathway (p = 0.043), and hematopoietic cell lineage (p = 0.048). The most upregulated KEGG pathways in the L group were retinol metabolism (p<0.001), nitrogen metabolism (p<0.001), drug metabolism - cytochrome P450 (p<0.001), metabolism of xenobiotics by cytochrome P450 (p<0.001), steroid hormone biosynthesis (p<0.001), bile secretion (p = 0.001), ascorbate and aldarate metabolism (p = 0.019), pentose and glucuronate interconversions (p = 0.019), tyrosine metabolism (p = 0.032), porphyrin metabolism (p = 0.032), glycine, serine and threonine metabolism (p = 0.032), fatty acid degradation (p = 0.032), pyruvate metabolism (p = 0.032), arginine and proline metabolism (p = 0.04), and chemical carcinogenesis - DNA adducts (p = 0.049). The most downregulated KEGG pathways in the L group were the IL-17 signaling pathway (p<0.001) and PPAR signaling pathway (p = 0.034).

### Effects of different dietary crude protein levels on the cecal microbial composition of weaning pigs

**Figure 2:**
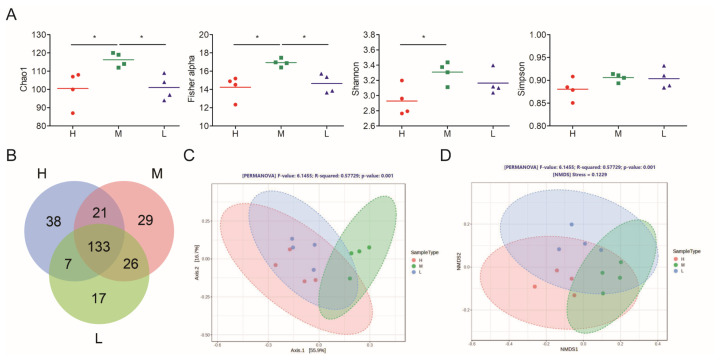
Microbial composition in the cecal digesta of weaning pigs fed diets containing different CP levels. (A) Microbial alpha diversity of cecal digesta. Each bar represents the mean (n = 4). * p<0.05. (B) Venn diagram for the distribution of microbiota (at the species level) in the cecal digesta. The value for each region represents the number of bacteria corresponding to the region. Results of the (C) principal coordinate analysis and (D) nonmetric multidimensional scaling of cecal bacterial communities at the species level. H: supplementation of 20% CP in the early phase (1–14 days) and 18% CP in the late phase (15–28 days); M: supplementation of 18% CP in the early phase and 16% CP in the late phase; L: supplementation of 16% CP in the early phase and 14% CP in the late phase. Data were analyzed from 4 replicate pens per treatment. CP, crude protein.

The effects of different dietary CP levels on the alpha diversity of cecal microbiota in weaning pigs at the end of the experiment are summarized in Figure 2A. Species richness, estimated using the Chao1 and Fisher alpha estimators, was greater in the M group than in the other groups (p≤0.05). The Shannon estimator, which indicates species evenness, was greater in the M group than in the H group (p≤0.05).

The Venn diagram in Figure 2B indicates considerable overlap (133 species, core) among the H, M, and L groups. Specifically, the H group had 38 unique bacterial types, the M group had 29, and the L group had 17. Shared bacterial types included 21 types between the H and M groups, 26 types between the M and L groups, and 7 types between the L and H groups. PCoA and NMDS suggested prominent between-group differences in cecal microbial composition (p = 0.001; Figures 2C, 2D).

The effects of different dietary CP levels on bacterial taxonomy in the cecal digesta of weaning pigs at the end of the experiment are summarized in Table 7. No significant between-group differences were observed in the relative abundance of bacteria at the phylum level. At the family level, Veillonellaceae was more abundant in the M and L groups than in the H group (p = 0.019 and p = 0.019, respectively). Lachnospiraceae was more abundant in the L group than in the H group (p = 0.05). At the genus level, *Limosilactobacillus* was more abundant in the H group than in the M group (p = 0.008). *Megasphaera* was more abundant in the M and L groups than in the H group (p = 0.019 and p = 0.019, respectively). *Blautia* was more abundant in the M and L groups than in the H group (p = 0.05 and p = 0.006, respectively). At the species level, *Lactobacillus johnsonii* and *Limosilactobacillus reuteri* subsp. *reuteri* were more abundant in the H group than in the M group (p = 0.011 and p = 0.003, respectively). *Megasphaera elsdenii* DSM 20460, *Blautia luti* DSM 14534, and *Dialister succinatiphilus* YIT 11850 were more abundant in the M group than in the H group (p = 0.019, p = 0.019, and p = 0.039, respectively). *L. johnsonii* was more abundant in the L group than in the M group (p = 0.031). *Lactobacillus amylovorus* DSM 20531 was less abundant in the H and L groups than in the M group (p≤0.05 and p = 0.006, respectively). *M. elsdenii* DSM 20460 was more abundant in the M group than in the L group (p = 0.019). *Mitsuokella jalaludinii* and *B. luti* DSM 14534 were more abundant in the L group than in the M group (p = 0.003 and p = 0.019, respectively).

A heatmap for the 50 most abundant species in the cecal digesta of weaning pigs (Supplement 2) revealed distinct clusters among the groups. The following species were most abundant in the cecal digesta of weaning pigs in the M and L groups: *Faecalibacterium duncaniae*, *Faecalibacterium prausnitzii*, *B. luti* DSM 14534, and *Streptococcus hyointestinalis*. The H and L groups shared 3 abundant species: *L. reuteri* subsp. *reuteri*, *L. johnsonii*, and *Eubacterium coprostanoligenes*. Two species were enriched in the M group: *L. amylovorus* DSM 20531 and *Lactobacillus rogosae*. A heatmap for microbial function in the cecal digesta of weaning pigs (Supplement 2) indicated that the groups differed in their functional profiles. The M group exhibited increased levels of functioning in secondary metabolite biosynthesis, global and overview maps, and amino acid metabolism and decreased levels of functioning in carbohydrate metabolism, membrane transport, cardiovascular disease, and transport and catabolism. The L group exhibited decreased levels of functioning in lipid metabolism, drug resistance (antineoplastic activity), nucleotide metabolism, and transcription and increased levels of functioning in translation, the immune system, the excretory system, the circulatory system, and DNA replication and repair.

### Associations between cecal bacterial species, growth performance, diarrhea incidence, fecal score, and cecal short-chain fatty acid level

**Figure 3:**
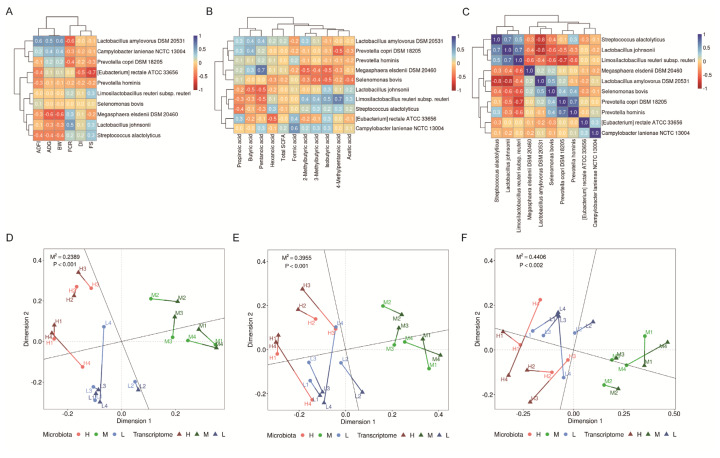
Correlations and integrated multi-omics analysis. (A) Correlation between growth performance and dominant bacteria. (B) Correlation between cecal short-chain fatty acid levels and dominant bacteria. (C) Correlations among dominant bacteria. (D) Procrustes analysis of the principal component analysis plot, (E) Procrustes analysis of the principal coordinate analysis plot, (F) Procrustes analysis of the nonmetric multidimensional scaling plot. Positive correlations are indicated in blue, whereas negative correlations are indicated in red. Correlation coefficients ranged from 1.0 to −1.0. H: supplementation of 20% CP in the early phase (1–14 days) and 18% CP in the late phase (15–28 days); M: supplementation of 18% CP in the early phase and 16% CP in the late phase; L: supplementation of 16% CP in the early phase and 14% CP in the late phase. Data were analyzed from 4 replicate pens per treatment. ADFI, average daily feed intake; ADG, average daily gain; BW, body weight; FCR, feed conversion ratio; DI, diarrhea incidence; FS, fecal score; CP, crude protein.

The average abundance of *L. amylovorus* DSM 20531 and *Campylobacter lanienae* NCTC 13004 in the cecal digesta was positively correlated with ADFI, ADG, and BW but negatively correlated with FCR (Figure 3A). The average abundance of *[Eubacterium] rectale* ATCC 33656 was negatively correlated with DI and FS (Figure 3A). The average abundance of *L. amylovorus* DSM 20531 was positively correlated with levels of butyric acid, pentanoic acid, and 2-methylbutyric acid in the cecal digesta (Figure 3B). Similarly, the average abundance of *C. lanienae* NCTC 13004 was positively correlated with the levels of pentanoic acid, formic acid, 3-methylbutyric acid, and isobutyric acid (Figure 3B). The average abundance of *L. amylovorus* DSM 20531 was positively correlated with that of *Selenomonas bovis*. Furthermore, the average abundance of *C. lanienae* NCTC 13004 was positively correlated with that of *[Eubacterium] rectale* ATCC 33656 (Figure 3C). Procrustes analysis, involving PCA, PCoA, and NMDS, revealed a strong correlation between microbial composition and transcriptome in the cecum of weaning pigs (p<0.001, p<0.001, and p = 0.002, respectively; Figures 3D–3F).

## DISCUSSION

Reducing dietary CP levels with amino acid supplementation in diets can help maintain growth performance to levels observed with diets formulated to meet the recommendations of the National Research Council [18]. A recent study showed that reduction in dietary CP concentrations decreased BW and ADG in weaning pigs [21]. Yu et al demonstrated a reduction in the growth performance of weaning pigs fed diets containing 14% CP compared with the performance of those fed diets containing 20% CP [22]. Reducing dietary CP levels from 18.5% to 16.6% in the early phase and 16.5% to 14.6% in the late phase resulted in reduced ADG and FCR [9]. Weaning pigs fed a low-CP diet (16% CP) exhibited poorer growth performance than did those fed a high-CP diet (20% CP) [6–8]. Reducing dietary CP levels from 19.79% to 17.08% has been demonstrated to limit the growth performance of weaning pigs [23]. However, no significant difference in growth performance was observed between diets containing 19% CP and those containing 22% CP [10]. Consistent with these findings, ours indicated that reducing dietary CP levels from 18% to 16% in the early phase and 16% to 14% in the late phase resulted in reduced BW, ADG, and FCR. However, no difference in growth performance was observed between pigs fed 18% CP in the early phase and 16% CP in the late phase and those fed 20% CP in the early phase and 18% CP in the late phase. Therefore, to optimize pig growth during the postweaning period, dietary CP levels should not be reduced below 18% in the early phase and below 16% in the late phase.

Pigs’ exposure to various stressors during the weaning period may affect their growth by shifting the use of dietary protein from growth to immune response or inflammation [24]. Excessive undigested protein in weaning pigs may be directed to the cecum for microbial fermentation, where it can be used by microbiota to produce toxic metabolites [15]. These toxic metabolites can impair gut integrity and function within the large intestine by inducing an inflammatory response [15]. Several cecal genes appear to be regulated in weaning pigs in response to diets containing different CP levels. Reducing dietary CP levels from 22% to 19% exerted no effects on the expression of tight junction and inflammation-related genes in the jejunum of weaning pigs [10]. However, a reduction in dietary CP from 18.5% to 16.5% altered the expression of amino acid transporters in the intestines of weaning pigs [8]. These findings suggest that reducing dietary CP levels to <16.5% regulates the expression of intestinal genes in weaning pigs. To the best of our knowledge, this study is the first to analyze changes in the cecal transcriptome of weaning pigs in response to diets containing different CP levels. Different dietary CP levels led to differential gene expression in the cecum of weaning pigs. Notably, the number of regulated genes in the M group compared with the H group were higher than in the L group compared with the H group. Several genes were upregulated in the M group relative to the H group, such as PLA2G2A and ANKRD34A. PLA2G2A is a secreted enzyme that hydrolyzes the sn-2 fatty acid acyl ester bond of phosphoglycerides, thereby liberating fatty acids and lysophospholipids [25]. In addition, PLA2G2A protects against gram-positive pathogenic bacteria by disrupting the bacterial cell wall [26]. Bacteria can induce the expression of PLA2G2A to eliminate competitor bacteria in the same niche and alter intestinal microbiota [27]. Therefore, PLA2G2A induced in the M group might have been released into the cecum’s lumen and modulated microbial composition through its antibacterial activity, thereby reducing the risk of gut pathogen infections; this explains why the pigs in the M group grew better than others. ANKRD34A belongs to ankyrin repeat-containing domain superfamily and member of the superfamily involves in cell-cell signaling, cytoskeleton integrity, transcription, inflammatory response, and development [28]. Dysfunction of ankyrin repeat-containing domain protein is implicated in numerous human diseases [28]. However, it is difficult to explain the function of induced ANKRD34A expression in the cecum of M group since the reports about the function of ANKRD34A is still scarce. The expression levels of C3orf85, SI, and COL11A2 were higher in the L group than in the H group. C3orf85 is involved in protein-coding processes; its mRNA expression level is higher in the colonocytes of patients with noninflammatory bowel disease than in those of patients with Crohn’s disease [29]. SI, an intestinal membrane-associated α-glucosidase, catalyzes carbohydrate digestion by breaking disaccharides and oligosaccharides into monosaccharides. The expression of SI is downregulated in the jejunum of pigs with colonic inflammation [30]. These findings explain why cecal inflammation (based on prediction of KEGG pathway) was lower in the L group than in the H group. Whether the upregulation of SI expression in the L group promoted the digestion of dietary carbohydrate in weaning pigs warrants further investigation. COL11A2, a minor fibrillar collagen, is present in the Golgi apparatus of normal colon goblet cells [31]. Deletion of COL11A2 gene in mice exhibit developmental defects, such as small body, deafness, and disorganized growth plate in long bones [32]. Whether the upregulation of COL11A2 expression in the L group contributed to the development of cecum remain to be confirmed. The expression levels of VSNL1, HOXD13, CA7, and MYOC were simultaneously reduced in the cecum of the M and L groups, indicating that these genes are sensitive to dietary CP levels. VSNL1, a calcium-sensor protein, plays multiple roles in dendritic growth, cyclic nucleotide signaling, and nicotinic modulation of neuronal network activity [33]. HOXD13 is a transcription factor that regulates differentiation and morphogenesis during animal development [34]. It promotes the progression of glioma by regulating tumor cell stemness, invasion, and growth [35]. CA7 is a cytosolic enzyme with high carbon dioxide hydration activity and inhibition of CA7 activity ameliorates inflammation in macrophages [36]. MYOC is a common pathogenic gene for primary open-angle glaucoma and olfactomedin domain of MYOC participates in protein-protein interactions, which are associated with inflammatory bowel disease [37]. Therefore, reducing the dietary level of CP can regulate the proliferation and differentiation of cecal epithelial cells and reduce the inflammation in the cecum. KEGG pathway analysis revealed significant reductions in the IL-17 signaling pathway in the M and L groups. IL-17 is a cytokine that contributes to the development of inflammatory bowel disease and exhibits strong proinflammatory activity [38]. Overall, the present study suggests that different dietary CP levels lead to differential gene expression patterns in the cecum of weaning pigs. Reducing dietary CP levels can alleviate inflammation in the cecum of weaning pigs, supporting the hypothesis that toxic metabolites derived from excessive undigested protein would induce gut inflammation.

Dietary CP levels strongly influence intestinal microbial composition and function. Protein–microbiota interactions play key roles in the health and growth of pigs [14]. The family Veillonellaceae is important groups of carbohydrate-degrading or fermenting microbes [39]. Reducing dietary CP levels (M and L groups) increased the abundance of the family Veillonellaceae in the cecal digesta of weaning pigs in the present study. Previous study reported a similar finding in cats, the abundance of the family Veillonellaceae was increased in the feces of low CP-fed kittens [40]. One possible explanation is that the ratio of dietary protein to carbohydrate was reduced, thereby increasing the growth of the Veillonellaceae species in the cecum of weaning pigs. Lachnospiraceae species are able to hydrolyze dietary carbohydrate to produce SCFA in the gut [41]. Dietary supplementation of 16% CP in the early phase and 14% CP in the late phase increased the abundance of the family Lachnospiraceae in the cecal digesta of weaning pigs. Similar to Veillonellaceae species, reduced dietary protein to carbohydrate ratio may promote the growth of the Lachnospiraceae species by utilizing dietary carbohydrate. *Megasphaera elsdenii* DSM 20460 and *Blautia luti* DSM 14534 belong to the genera *Megasphaera* (family Veillonellaceae) and *Blautia* (family Lachnospiraceae). *Megasphaera elsdenii* and *Blautia luti* were characterized as an anaerobic carbohydrate fermenter [42,43]. These two species were simultaneously increased in the cecal digesta of the M and L groups, indicating that these species are sensitive to dietary CP levels and may prefer to metabolize carbohydrates in a low CP diet.

Procrustes analysis in the present study revealed a highly significant correlation between microbial composition and transcriptome in the cecum of weaning pigs, demonstrating that interactions between dietary CP levels and cecal microbiota are crucial for cecal health. Dietary supplementation of 18% CP in the early phase and 16% CP in the late phase resulted in the optimal growth performance. This dietary regimen reduced the abundance of *L. johnsonii* and increased that of *L. amylovorus* DSM 20531 in the cecal digesta, suggesting that the ratio of *L. johnsonii* to *L. amylovorus* DSM 20531 influences the maintenance of intestinal function and growth in weaning pigs. Furthermore, the abundance of *L. amylovorus* DSM 20531 was positively correlated with the level of SCFA in the cecum. SCFAs regulate energy metabolism, cell growth, and inflammation in the gut [44]. Inconsistent results have been reported regarding the effects of a low-CP diet on the abundance of the genus *Lactobacillus* in the gut of weaning pigs [45]. Our study clarified which *Lactobacillus* sp. is sensitive to dietary CP levels in the cecum and associated changes in the abundance of *Lactobacillus* sp. with the growth performance of weaning pigs. Overall, reducing dietary CP levels can alter cecal microbial composition, SCFA levels, and cecal gene expression, ultimately affecting the growth of weaning pigs.

A lower villus height to crypt depth ratio in the ileum of weaning pigs fed low CP diet (16% in the early phase and 14% in the late phase) supplemented with essential amino acids was observed in the present study. A higher villus height to crypt depth ratio can promote the efficiency of nutrient absorption, thereby improving FCR of weaning pigs. Supplementation of branched-chain amino acid (BCAA) to low CP diets can restore FCR of weaning pigs [46]. BCAAs are essential for maintaining intestinal mucosal integrity and exert anti-inflammatory effects in intestinal cells exposed to lipopolysaccharide [47]. Therefore, the poor FCR in the L group is likely due to BCAA deficiency-mediated impaired gut morphology in a low CP diet. Cecal PPAR signaling pathway was downregulated and the abundance of *L. amylovorus* DSM 20531 was reduced in the cecal digesta of L group. PPAR mitigates intestinal barrier dysfunction and inflammation induced by pathogens [48]. These findings further explained the poor FCR in the L group compared with the other groups.

## CONCLUSION

This study provides preliminary evidence for the differential effects of different dietary CP levels on the growth performance, cecal transcriptome, and cecal microbial composition of weaning pigs. Reducing the CP level in the diet of weaning pigs modulate cell growth in the cecum by downregulation of cell proliferation and differentiation-associated genes. CP reduction has beneficial effects on the anti-inflammation in the cecum by the reduction of inflammation-associated gene expression. Reducing the level of CP alters cecal microbiota composition and promotes the growth of carbohydrate-fermenting bacteria. Microbial composition was significantly correlated with transcriptome profiles in the cecum. Compared with other dietary regimens, 18% CP in the early phase and 16% CP in the late phase markedly improved growth and gut health in weaning pigs.

## Figures and Tables

**Table 1:** Compositions of the 3 experimental diets

Item	H^1)^		M		L	

	1–14 d	15–28 d	1–14 d	15–28 d	1–14 d	15–28 d
Ingredient (%)						
Corn, yellow, 6.9% CP	57.9	63.07	59.51	66.11	63.41	68.76
Soybean meal, 43% CP	23.0	20.2	21.5	16.6	17.0	16.0
Fish meal, 63% CP	10.0	8.0	8.0	7.0	7.0	4.0
Dried whey, 13% CP	4.4	4.0	4.0	3.5	3.5	3.5
Soybean oil	4.0	3.6	4.45	4.15	5.0	4.2
CaCO_3_, 38% Ca	0	0	0.86	0.85	1.72	1.32
CaHPO_4_	0	0	0.31	0.35	0.66	0.44
Salt	0.3	0.3	0.3	0.3	0.3	0.3
Choline, 50%	0.12	0.12	0.12	0.12	0.12	0.12
Vitamin, premix^2)^	0.1	0.1	0.1	0.1	0.1	0.1
Mineral, premix^3)^	0.1	0.1	0.1	0.1	0.1	0.1
Lysine, 99%	0.24	0.27	0.38	0.43	0.56	0.60
Methionine	0.11	0.11	0.15	0.14	0.18	0.20
Threonine	0.13	0.13	0.20	0.22	0.30	0.31
Tryptophan	0	0	0.02	0.03	0.05	0.05
Chemical composition (%)^4)^						
Crude protein	20.0	18.1	18.0	15.9	15.9	14.2
Calcium	2.9	2.57	2.9	2.57	2.9	2.57
Phosphorus	3.3	2.99	3.3	2.99	3.3	2.99
Lysine	1.54	1.41	1.54	1.41	1.54	1.41
Methionine	0.45	0.41	0.45	0.41	0.45	0.41
Threonine	0.96	0.88	0.96	0.88	0.96	0.88
Tryptophan	0.27	0.25	0.27	0.25	0.27	0.25
Isoleucine	0.91	0.81	0.83	0.71	0.71	0.61
Histidine	0.49	0.44	0.45	0.40	0.40	0.35
Phenyalanine	0.95	0.86	0.88	0.76	0.76	0.68
Leucine	1.86	1.70	1.72	1.53	1.53	1.38
Valine	1.14	1.02	1.04	0.90	0.90	0.78
Aspartic acid	2.07	1.84	1.89	1.61	1.62	1.41
Serine	0.86	0.77	0.79	0.68	0.69	0.61
Glutamic acid	3.29	3.02	3.06	2.70	2.07	2.45
Proline	1.26	1.17	1.18	1.06	1.05	0.98
Glycine	1.03	0.91	0.92	0.80	0.80	0.66
Alanine	1.13	1.02	1.03	0.92	0.92	0.80
Cysteine	0.15	0.13	0.13	0.11	0.11	0.10
Tyrosine	0.59	0.53	0.54	0.47	0.47	0.41
Arginine	1.28	1.14	1.17	1.01	1.01	0.88
ME (kcal/kg)	3,400	3,400	3,400	3,400	3,400	3,400

1) H: supplementation of 20% crude protein (CP) in the early phase (1–14 days) and 18% CP in the late phase (15–28 days); M: supplementation of 18% CP in the early phase and 16% CP in the late phase; L: supplementation of 16% CP in the early phase and 14% CP in the late phase.

2) Vitamin premix provided the following (per kilogram of diet): 6,000 IU of vitamin A; 900 IU of vitamin D3; 30 IU of vitamin E; 3 mg of menadione (to provide vitamin K); 6 mg of riboflavin; 18 mg of d-pantothenic acid; 60 mg of niacin; and 0.03 mg of vitamin B12.

3) Trace mineral premix provided the following (per kilogram of diet): 20 mg of Cu (copper sulfate); 100 mg of Zn (zinc sulfate); 140 mg of Fe (ferrous sulfate); 40 mg of Mn (manganese sulfate); 0.2 mg of I (calcium iodate); and 0.1 mg of Se (sodium selenite).

4) Crude protein is analyzed and others are calculated values.

**Table 2:** Effects of different dietary CP levels on the growth performance, fecal score, and diarrhea incidence of weaning pigs

	H^1)^	M	L	SEM	p-value
Body weight (kg/head)					
1 d	7.6^2)^	7.6	7.6	0.01	0.990
28 d	16.0^a^	16.4^a^	14.6^b^	0.29	0.008
Average daily gain (kg/d/head)					
1–14 d	0.19	0.18	0.14	0.01	0.058
15–28 d	0.41^ab^	0.45^a^	0.36^b^	0.02	0.014
1–28 d	0.30^a^	0.31^a^	0.25^b^	0.01	0.008
Average daily feed intake (kg/d/head)					
1–14 d	0.37	0.36	0.35	0.01	0.483
15–28 d	0.63	0.67	0.58	0.02	0.168
1–28 d	0.50	0.51	0.47	0.01	0.222
Feed conversion ratio					
1–14 d	1.9^b^	2.1^ab^	2.5^a^	0.11	0.046
15–28 d	1.6	1.5	1.6	0.04	0.282
1–28 d	1.7^b^	1.6^b^	1.9^a^	0.04	0.010
Mortality (%)					
1–28 d	3.1	9.4	9.4	2.41	0.519
Fecal score					
1–14 d	2.5	2.4	2.2	0.07	0.081
15–28 d	2.6	2.2	2.2	0.08	0.123
1–28 d	2.6	2.3	2.2	0.07	0.108
Diarrhea incidence (%)					
1–14 d	21.9	22.0	13.8	2.60	0.371
15–28 d	19.9	16.5	19.5	2.43	0.853
1–28 d	20.9	19.3	16.7	2.06	0.740

1) H: supplementation of 20% CP in the early phase (1–14 days) and 18% CP in the late phase (15–28 days); M: supplementation of 18% CP in the early phase and 16% CP in the late phase; L: supplementation of 16% CP in the early phase and 14% CP in the late phase.

2) Data are expressed in terms of mean (n = 4) values.

a,b In the rows, mean values without common superscripts indicate significant changes (p≤0.05).

CP, crude protein; SEM, standard error of the mean.

**Table 3:** Effects of different dietary CP levels on the blood biochemistry of weaning pigs

	H^1)^	M	L	SEM	p-value
Glucose (mg/dL)	105.5^2)^	93.9	98.3	3.32	0.388
Triglyceride (mg/dL)	50.25	65.1	63.8	3.43	0.147
Cholesterol (mg/dL)	74.5	77.3	76.8	1.37	0.723
High-density lipoprotein (mg/dL)	25.4	26.9	26.4	0.88	0.809
Low-density lipoprotein (mg/dL)	33.5	35.3	34.6	1.08	0.828
Aspartate transaminase (U/L)	85.0^a^	75.3^ab^	65.5^b^	3.15	0.020
Alanine aminotransferase (U/L)	63.8	66.9	61.4	2.91	0.777
Alkaline phosphatase (U/L)	217.6	211.0	205.4	11.97	0.930
Total protein (g/dL)	5.4	5.4	5.3	0.09	0.946
Albumin (g/dL)	3.0	3.0	2.9	0.04	0.620
Globulin (g/dL)	2.4	2.4	2.4	0.09	0.975
Blood urea nitrogen (mg/dL)	6.1^a^	4.2^ab^	3.4^b^	0.47	0.032
Creatinine (mg/dL)	0.9	0.9	0.8	0.02	0.286
Cortisol (μg/dL)	3.6^a^	2.0^ab^	0.9^b^	0.42	0.007

1) H: supplementation of 20% CP in the early phase (1–14 days) and 18% CP in the late phase (15–28 days); M: supplementation of 18% CP in the early phase and 16% CP in the late phase; L: supplementation of 16% CP in the early phase and 14% CP in the late phase.

2) Data are expressed in terms of mean (n = 4) values.

a,b In the rows, mean values without common superscripts indicate significant changes (p≤0.05).

CP, crude protein; SEM, standard error of the mean.

**Table 4:** Effects of different dietary CP levels on the gut morphology of weaning pigs

		H^1)^	M	L	SEM	p-value
Duodenum	Villus height (μm)	452.5^2),ab^	521.7^a^	379.7^b^	20.73	0.004
	Crypt depth (μm)	314.4	310.6	375.3	13.55	0.077
	Villus height: Crypt depth	1.5^a^	1.8^a^	1.0^b^	0.10	<0.001
Jejunum	Villus height (μm)	427.2^ab^	475.0^a^	363.3^b^	16.25	0.003
	Crypt depth (μm)	279.2	255.8	300.0	10.84	0.271
	Villus height: Crypt depth	1.7	2.1	1.3	0.17	0.140
Ileum	Villus height (μm)	394.4	395.3	325.8	14.79	0.074
	Crypt depth (μm)	247.5	219.4	261.1	9.38	0.187
	Villus height: Crypt depth	1.7^a^	1.9^a^	1.3^b^	0.08	0.003

1) H: supplementation of 20% CP in the early phase (1–14 days) and 18% CP in the late phase (15–28 days); M: supplementation of 18% CP in the early phase and 16% CP in the late phase; L: supplementation of 16% CP in the early phase and 14% CP in the late phase.

2) Data are expressed in terms of mean (n = 4) values.

a,b In the rows, mean values without common superscripts indicate significant changes (p≤0.05).

CP, crude protein; SEM, standard error of the mean.

**Table 5:** Ten most regulated genes in the cecum of weaning pigs in response to different dietary CP levels

	Regulation	Ensemble gene ID	Gene symbol	Description	Fold change	p-value^1)^
M versus H^2)^	Up	00000003494	*PLA2G2A*	Phospholipase A2 group IIA	10.1	<0.001
		00000037260	Unknown	Unknown	5.6	<0.001
		00000050531	Unknown	Unknown	3.8	<0.001
		00000037823	*ANKRD34A*	Ankyrin repeat domain 34A	3.8	0.002
		00000049869	Unknown	Unknown	3.6	0.021
		00000007488	*DOK5*	Docking protein 5	3.6	0.019
		00000032588	*C3orf85*	Chromosome 3 open reading frame 85	3.3	<0.001
		00000051144	Unknown	Unknown	3.1	0.001
		00000046357	Unknown	Unknown	3.1	0.022
		00000011217	*NEK10*	NIMA related kinase 10	3.1	0.046
	Down	00000008615	*VSNL1*	Visinin like 1	−7.2	<0.001
		00000015979	*HOXD13*	Homeobox D13	−7.0	<0.001
		00000021938	*UPP2*	Uridine phosphorylase 2	−5.2	0.001
		00000049086	Unknown	Unknown	−5.1	<0.001
		00000035066	*MYOC*	Myocilin	−4.8	<0.001
		00000013060	*SCGB1A1*	Secretoglobin family 1A member 1	−4.5	0.002
		00000032826	*KCTD16*	Potassium channel tetramerization domain containing 16	−4.4	0.003
		00000000871	*SLC5A8*	Solute carrier family 5 member 8	−4.2	<0.001
		00000038785	*CA7*	Carbonic anhydrase 7	−4.1	<0.001
		00000047906	Unknown	Unknown	−3.9	<0.001
L versus H	Up	00000032588	*C3orf85*	Chromosome 3 open reading frame 85	3.4	<0.001
		00000046777	Unknown	Unknown	2.4	<0.001
		00000042459	Unknown	Unknown	2.2	<0.001
		00000049575	Unknown	Unknown	2.2	<0.001
		00000011735	*SI*	Sucrase–isomaltase	2.0	0.009
		00000039341	Unknown	Unknown	1.9	<0.001
		00000028627	Unknown	Unknown	1.9	<0.001
		00000001473	*COL11A2*	Collagen type XI alpha 2 chain	1.9	0.004
		00000047669	Unknown	Unknown	1.9	0.002
		00000023607	*CYP4F22*	Cytochrome P450 family 4 subfamily F member 22	1.8	<0.001
	Down	00000015979	*HOXD13*	Homeobox D13	−6.8	<0.001
		00000008615	*VSNL1*	Visinin like 1	−5.5	<0.001
		00000045751	Unknown	Unknown	−3.7	<0.001
		00000039573	*SLPI*	Secretory leukocyte peptidase inhibitor	−3.7	<0.001
		00000038785	*CA7*	Carbonic anhydrase 7	−3.4	<0.001
		00000009561	*ATP4B*	Potassium-transporting ATPase subunit beta	−3.0	<0.001
		00000033015	*B3GNT7*	betaGal beta-1,3-N-acetylglucosaminyltransferase 7	−2.9	<0.001
		00000028758	*LBP*	Lipopolysaccharide-binding protein	−2.9	<0.001
		00000007384	*WFDC5*	WAP four-disulfide core domain 5	−2.9	<0.001
		00000035066	*MYOC*	Myocilin	−2.8	<0.001

1) Adjusted p-value: false discovery rate was corrected using the Benjamini–Hochberg method.

2) H: supplementation of 20% CP in the early phase (1–14 days) and 18% CP in the late phase (15–28 days); M: supplementation of 18% CP in the early phase and 16% CP in the late phase; L: supplementation of 16% CP in the early phase and 14% CP in the late phase.

CP, crude protein.

**Table 6:** Most modulated Kyoto Encyclopedia of Genes and Genomes pathways in the cecum of weaning pigs in response to different dietary CP levels

	Regulation	Pathway	p-value^1)^
M versus H^2)^	Up	Cytoskeleton in muscle cells	<0.001
		cAMP signaling pathway	<0.001
		Neuroactive ligand–receptor interaction	<0.001
		Calcium signaling pathway	<0.001
		Adrenergic signaling in cardiomyocytes	<0.001
		Motor proteins	<0.001
		Vascular smooth muscle contraction	<0.001
		Cardiac muscle contraction	<0.001
		Axon guidance	0.002
		Nicotine addiction	0.006
		Dilated cardiomyopathy	0.016
		Glutamatergic synapse	0.025
		Insulin secretion	0.025
		Circadian entrainment	0.047
		Hypertrophic cardiomyopathy	0.047
	Down	IL-17 signaling pathway	0.027
		Cytosolic DNA-sensing pathway	0.043
		TNF signaling pathway	0.043
		Hematopoietic cell lineage	0.048
L versus H	Up	Retinol metabolism	<0.001
		Nitrogen metabolism	<0.001
		Drug metabolism - cytochrome P450	<0.001
		Metabolism of xenobiotics by cytochrome P450	<0.001
		Steroid hormone biosynthesis	<0.001
		Bile secretion	0.001
		Ascorbate and aldarate metabolism	0.019
		Pentose and glucuronate interconversions	0.019
		Tyrosine metabolism	0.032
		Porphyrin metabolism	0.032
		Glycine, serine and threonine metabolism	0.032
		Fatty acid degradation	0.032
		Pyruvate metabolism	0.032
		Arginine and proline metabolism	0.040
		Chemical carcinogenesis - DNA adducts	0.049
	Down	IL-17 signaling pathway	<0.001
		PPAR signaling pathway	0.034

1) Adjusted p-value: false discovery rate was corrected using the Benjamini–Hochberg method.

2) H: supplementation of 20% CP in the early phase (1–14 days) and 18% CP in the late phase (15–28 days); M: supplementation of 18% CP in the early phase and 16% CP in the late phase; L: supplementation of 16% CP in the early phase and 14% CP in the late phase.

CP, crude protein.

**Table 7:** Effects of different dietary CP levels on the cecal bacterial taxonomy of weaning pigs

	H^1)^	M	L	SEM	p-value		

					H vs. M	M vs. L	L vs. H
Phylum							
Firmicutes	90.6^2)^	83.9	88.1	1.87	0.239	0.378	0.769
Bacteroidota	6.3	11.4	8.9	1.41	0.170	0.695	0.163
Family							
Lactobacillaceae	52.4	40.7	39.4	2.62	0.141	0.769	0.078
Veillonellaceae	7.3	11.9	11.4	0.90	0.019	1.000	0.019
Streptococcaceae	11.2	7.9	11.5	1.17	0.239	0.239	1.000
Lachnospiraceae	8.5	9.6	11.9	0.61	0.493	0.202	0.050
Prevotellaceae	5.8	10.8	8.8	1.36	0.239	0.769	0.378
Oscillospiraceae	3.9	4.3	4.4	0.27	0.556	0.769	0.769
Genus							
*Lactobacillus*	38.3	33.7	30.9	1.65	0.433	0.433	0.117
*Streptococcus*	11.2	7.9	11.5	1.17	0.239	0.239	1.000
*Limosilactobacillus*	13.7	6.2	8.2	1.18	0.008	0.378	0.078
*Megasphaera*	6.3	10.2	9.9	0.85	0.019	1.000	0.019
*Prevotella*	4.4	8.2	7.1	1.04	0.117	0.845	0.170
*Blautia*	1.6	2.6	3.1	0.23	0.050	0.433	0.006
*Campylobacter*	2.1	2.0	1.7	0.37	0.695	0.695	0.433
*Selenomonas*	1.2	2.2	1.7	0.29	0.117	0.433	0.433
*Eubacterium*	1.5	1.2	2.1	0.25	0.767	0.239	0.378
*Prevotellamassilia*	1.2	2.3	1.2	0.38	0.096	0.493	0.327
Species							
*Lactobacillus johnsonii*	25.2	6.4	22.6	2.73	0.011	0.031	0.695
*Lactobacillus amylovorus* DSM 20531	11.1	25.0	6.9	2.73	0.050	0.006	0.433
*Streptococcus alactolyticus*	11.1	6.2	10.8	1.28	0.050	0.117	0.695
*Megasphaera elsdenii* DSM 20460	6.3	10.2	9.8	0.85	0.019	1.000	0.019
*Limosilactobacillus reuteri subsp. reuteri*	11.7	4.1	7.4	1.05	0.003	0.239	0.078
*Prevotella copri* DSM 18205	1.9	3.9	3.0	0.48	0.062	0.281	0.433
*Campylobacter lanienae* NCTC 13004	2.1	2.0	1.7	0.37	0.695	0.695	0.433
*Prevotella hominis*	1.3	1.9	2.1	0.27	0.327	0.922	0.281
*Selenomonas bovis*	1.2	2.2	1.7	0.29	0.117	0.433	0.433
*[Eubacterium] rectale* ATCC 33656	1.5	1.2	2.1	0.25	0.769	0.239	0.378
*Prevotellamassilia timonensis*	1.2	2.3	1.2	0.38	0.096	0.327	0.493
*Mitsuokella jalaludinii*	0.4	1.3	2.5	0.34	0.078	0.239	0.003
*Limosilactobacillus mucosae*	1.8	1.6	0.7	0.27	0.845	0.062	0.096
*Blautia luti* DSM 14534	0.7	1.5	1.7	0.16	0.019	1.000	0.019
*Dialister succinatiphilus* YIT 11850	0.9	1.5	1.3	0.10	0.039	0.769	0.078

1) H: supplementation of 20% CP in the early phase (1–14 days) and 18% CP in the late phase (15–28 days); M: supplementation of 18% CP in the early phase and 16% CP in the late phase; L: supplementation of 16% CP in the early phase and 14% CP in the late phase.

2) Data are expressed in terms of mean (n = 4) values.

CP, crude protein; SEM, standard error of the mean.

## SUPPLEMENTARY MATERIAL

**Supplement 1.** Effects of different dietary CP levels on the cecal short-chain fatty acid levels of weaning pigs.

**Supplement 2.** Heatmap for bacterial distribution and microbial function.

## References

[1] Jensen MS Jensen SK Jakobsen K Development of digestive enzymes in pigs with emphasis on lipolytic activity in the stomach and pancreas J Anim Sci 1997 75 437 45 10.2527/1997.752437x 9051467

[2] Heo JM Kim JC Hansen CF Mullan BP Hampson DJ Pluske JR Effects of feeding low protein diets to piglets on plasma urea nitrogen, faecal ammonia nitrogen, the incidence of diarrhoea and performance after weaning Arch Anim Nutr 2008 62 343 58 10.1080/17450390802327811 18942582

[3] Heo JM Kim JC Hansen CF Effects of dietary protein level and zinc oxide supplementation on the incidence of post-weaning diarrhoea in weaner pigs challenged with an enterotoxigenic strain of Escherichia coli Livest Sci 2010 133 210 3 10.1016/j.livsci.2010.06.066

[4] Opapeju FO Rademacher M Blank G Nyachoti CM Effect of low-protein amino acid-supplemented diets on the growth performance, gut morphology, organ weights and digesta characteristics of weaned pigs Animal 2008 2 1457 64 10.1017/S175173110800270X 22443903

[5] Opapeju FO Krause DO Payne RL Rademacher M Nyachoti CM Effect of dietary protein level on growth performance, indicators of enteric health, and gastrointestinal microbial ecology of weaned pigs induced with postweaning colibacillosis J Anim Sci 2009 87 2635 43 10.2527/jas.2008-1310 19395520

[6] Hou L Wang L Qiu Y Effects of protein restriction and subsequent realimentation on body composition, gut microbiota and metabolite profiles in weaned piglets Animals 2021 11 686 10.3390/ani11030686 33806535 PMC8001264

[7] Wellington MO Hulshof TG Resink JW The effect of supplementation of essential amino acid combinations in a low crude protein diet on growth performance in weanling pigs Transl Anim Sci 2023 7 txad008 10.1093/tas/txad008 36777099 PMC9909505

[8] Lin Q Tu X Li X Effects of low protein diets on acid-base balance, electrolyte balance, intestinal structure, and amino acid transport in piglets J Anim Physiol Anim Nutr 2024 108 1107 18 10.1111/jpn.13954 38567963

[9] Marchetti R Faeti V Gallo M Protein content in the diet influences growth and diarrhea in weaning piglets Animals 2023 13 795 10.3390/ani13050795 36899653 PMC10000050

[10] Lee J González-Vega JC Htoo JK Effects of dietary protein content and crystalline amino acid supplementation patterns on growth performance, intestinal histomorphology, and immune response in weaned pigs raised under different sanitary conditions J Anim Sci 2022 100 skac285. 36062846 10.1093/jas/skac285 PMC9527300

[11] Stumpff F Manneck D Martens H Unravelling the secrets of the caecum Pflugers Arch 2019 471 925 6 10.1007/s00424-019-02292-1 31197447

[12] Allison MJ Robinson IM Bucklin JA Booth GD Comparison of bacterial populations of the pig cecum and colon based upon enumeration with specific energy sources Appl Environ Microbiol 1979 37 1142 51 10.1128/aem.37.6.1142-1151.1979 384906 PMC243369

[13] Sun Y Su Y Zhu W Microbiome-metabolome responses in the cecum and colon of pig to a high resistant starch diet Front Microbiol 2016 7 779 10.3389/fmicb.2016.00779 27303373 PMC4880592

[14] Blachier F Andriamihaja M Kong XF Fate of undigested proteins in the pig large intestine: what impact on the colon epithelium? Anim Nutr 2021 9 110 8 10.1016/j.aninu.2021.08.001 35573094 PMC9065739

[15] Xia J Fan H Yang J Research progress on diarrhoea and its mechanism in weaned piglets fed a high-protein diet J Anim Physiol Anim Nutr 2022 106 1277 87 10.1111/jpn.13654 34719816

[16] Wellock IJ Fortomaris PD Houdijk JGM Kyriazakis I Effects of dietary protein supply, weaning age and experimental enterotoxigenic Escherichia coli infection on newly weaned pigs: health Animal 2008 2 834 42 10.1017/S1751731108002048 22443662

[17] Gao J Yin J Xu K Protein level and infantile diarrhea in a postweaning piglet model Mediators Inflamm 2020 2020 937387 10.1155/2020/1937387 PMC7281817 32565721

[18] National Research Council Nutrient requirements of swine 11th ed The National Academies Press 2012

[19] Official Methods of Analysis 22nd ed Association of Official Analytical Chemists International 2005

[20] Cheng YH Su LW Horng YB Yu YH Effects of soybean meal fermented by Lactobacillus species and Clostridium butyricum on growth performance, diarrhea incidence, and fecal bacteria in weaning piglets Ann Anim Sci 2019 19 1051 62 10.2478/aoas-2019-0042

[21] Cho I Kong C Growth performance of pigs fed low-protein diets supplemented with crystalline amino acids at different growth stages Anim Biosci 2025 38 316 24 10.5713/ab.24.0339 39210807 PMC11725741

[22] Yu D Zhu W Hang S Effects of low-protein diet on the intestinal morphology, digestive enzyme activity, blood urea nitrogen, and gut microbiota and metabolites in weaned pigs Arch Anim Nutr 2019 73 287 305 10.1080/1745039X.2019.1614849 31163993

[23] Hu N Shen Z Pan L Qin G Zhao Y Bao N Effects of protein content and the inclusion of protein sources with different amino acid release dynamics on the nitrogen utilization of weaned piglets Anim Biosci 2022 35 260 71 10.5713/ab.21.0142 34445847 PMC8738945

[24] Zhang H Wielen NV Hee BV Wang J Hendriks W Gilbert M Impact of fermentable protein, by feeding high protein diets, on microbial composition, microbial catabolic activity, gut health and beyond in pigs Microorganisms 2020 8 1735 10.3390/microorganisms8111735 33167470 PMC7694525

[25] van Hensbergen VP Wu Y van Sorge NM Touqui L Type IIA secreted phospholipase A2 in host defense against bacterial Infections Trends Immunol 2020 41 313 26 10.1016/j.it.2020.02.003 32151494

[26] Koprivnjak T Peschel A Gelb MH Liang NS Weiss JP Role of charge properties of bacterial envelope in bactericidal action of human group IIA phospholipase A2 against Staphylococcus aureus J Biol Chem 2002 277 47636 44 10.1074/jbc.M205104200 12359734

[27] Doré E Joly-Beauparlant C Morozumi S The interaction of secreted phospholipase A2-IIA with the microbiota alters its lipidome and promotes inflammation JCI Insight 2022 7 e152638 10.1172/jci.insight.152638 35076027 PMC8855825

[28] Chagula DB Rechciński T Rudnicka K Chmiela M Ankyrins in human health and disease - an update of recent experimental findings Arch Med Sci 2019 16 715 26 10.5114/aoms.2019.89836 32542072 PMC7286341

[29] Kanke M Kennedy Ng MM Connelly S Single-cell analysis reveals unexpected cellular changes and transposon expression signatures in the colonic epithelium of treatment-naïve adult Crohn’s disease patients Cell Mol Gastroenterol Hepatol 2022 13 1717 40 10.1016/j.jcmgh.2022.02.005 35158099 PMC9046244

[30] Lackeyram D Mine Y Archbold T Fan MZ The small intestinal apical hydrolase activities are decreased in the piglet with bowel inflammation induced by dextran sodium sulfate J Anim Sci 2012 90 Suppl 4 287 9 10.2527/jas.54010 23365358

[31] Bowen KB Reimers AP Luman S Kronz JD Fyffe WE Oxford JT Immunohistochemical localization of collagen type XI alpha1 and alpha2 chains in human colon tissue J Histochem Cytochem 2008 56 275 83 10.1369/jhc.7A7310.2007 18040076 PMC2324180

[32] Li SW Takanosu M Arita M Targeted disruption of Col11a2 produces a mild cartilage phenotype in transgenic mice: comparison with the human disorder otospondylomegaepiphyseal dysplasia (OSMED) Dev Dyn 2001 222 141 52 10.1002/dvdy.1178 11668593

[33] Braunewell KH The visinin-like proteins VILIP-1 and VILIP-3 in Alzheimer’s disease-old wine in new bottles Front Mol Neurosci 2012 5 20 10.3389/fnmol.2012.00020 22375104 PMC3284765

[34] Muragaki Y Mundlos S Upton J Olsen BR Altered growth and branching patterns in synpolydactyly caused by mutations in HOXD13 Science 1996 272 548 51 10.1126/science.272.5261.548 8614804

[35] Zhang J Deng M Tong H A novel miR-7156-3p-HOXD13 axis modulates glioma progression by regulating tumor cell stemness Int J Biol Sci 2020 16 3200 9 10.7150/ijbs.51293 33162825 PMC7645993

[36] Hudalla H Michael Z Christodoulou N Carbonic anhydrase inhibition ameliorates inflammation and experimental pulmonary hypertension Am J Respir Cell Mol Biol 2019 61 512 24 10.1165/rcmb.2018-0232OC 30951642 PMC6775956

[37] Hill SE Donegan RK Nguyen E Desai TM Lieberman RL Molecular details of olfactomedin domains provide pathway to structure-function studies PLOS ONE 2015 10 e0130888 10.1371/journal.pone.0130888 26121352 PMC4488277

[38] Magyari L Kovesdi E Sarlos P Javorhazy A Sumegi K Melegh B Interleukin and interleukin receptor gene polymorphisms in inflammatory bowel diseases susceptibility World J Gastroenterol 2014 20 3208 22 10.3748/wjg.v20.i12.3208 24695754 PMC3964393

[39] Vahidi MF Gharechahi J Behmanesh M Ding XZ Han JL Hosseini Salekdeh G Diversity of microbes colonizing forages of varying lignocellulose properties in the sheep rumen PeerJ 2021 9 e10463 10.7717/peerj.10463 33510967 PMC7808268

[40] Hooda S Vester Boler BM Kerr KR Dowd SE Swanson KS The gut microbiome of kittens is affected by dietary protein: carbohydrate ratio and associated with blood metabolite and hormone concentrations Br J Nutr 2013 109 1637 46 10.1017/S0007114512003479 22935193

[41] Biddle A Stewart L Blanchard J Leschine S Untangling the genetic basis of fibrolytic specialization by Lachnospiraceae and Ruminococcaceae in diverse gut communities Diversity 2013 5 627 40 10.3390/d5030627

[42] Cabral LDS Weimer PJ Megasphaera elsdenii: its role in ruminant nutrition and its potential industrial application for organic acid biosynthesis Microorganisms 2024 12 219 10.3390/microorganisms12010219 38276203 PMC10819428

[43] Liu X Mao B Gu J Wu J Blautia-a new functional genus with potential probiotic properties? Gut Microbes 2021 13 1 21 10.1080/19490976.2021.1875796 PMC7872077 33525961

[44] den Besten G van Eunen K Groen AK Venema K Reijngoud DJ Bakker BM The role of short-chain fatty acids in the interplay between diet, gut microbiota, and host energy metabolism J Lipid Res 2013 54 2325 40 10.1194/jlr.R036012 23821742 PMC3735932

[45] Sadurní M Barroeta AC Sol C Puyalto M Castillejos L Effects of dietary crude protein level and sodium butyrate protected by medium-chain fatty acid salts on performance and gut health in weaned piglets J Anim Sci 2023 101 skad090 10.1093/jas/skad090 36967519 PMC10103067

[46] Garcia BREV Makiyama EN Sampaio GR Effects of branched-chain amino acids on the inflammatory response induced by LPS in Caco-2 Cells Metabolites 2024 14 76 10.3390/metabo14010076 38276311 PMC10821323

[47] Zheng L Wei H Cheng C Xiang Q Pang J Peng J Supplementation of branched-chain amino acids to a reduced-protein diet improves growth performance in piglets: involvement of increased feed intake and direct muscle growth-promoting effect Br J Nutr 2016 115 2236 45 10.1017/S0007114516000842 27079773

[48] Noori M Azimirad M Ghorbaninejad M Meyfour A Zali MR Yadegar A PPAR-γ agonist mitigates intestinal barrier dysfunction and inflammation induced by Clostridioides difficile SlpA in vitro Sci Rep 2024 14 32087 10.1038/s41598-024-83815-4 39738433 PMC11686163

