# Supplementary material for: Transcriptome and microbiota analysis reveal differences in the cecum of weaning pigs in response to different dietary crude protein levels
Source: Anim Biosci. 2025 Aug 12;39(1):250135. doi: 10.5713/ab.25.0135 (PMC12754465; doi:10.5713/ab.25.0135)
Supplement: Supplementary file 1 [file ab-25-0135-Supplementary-1.pdf]

Supplement 1. Effects of different dietary CP levels on the cecal short-chain fatty acid levels of weaning pigs.

	H[1)]	M	L	SEM	p value
Formic acid (μg/mg[3)])	46.3[2)]	25.6	53.5	7.19	0.282
Acetic acid (μg/mg)	2964.7	3108.3	2890.1	198.18	0.917
Propionic acid (μg/mg)	1624.6	2265.3	2296.3	174.76	0.221
Butyric acid (μg/mg)	163.8	277.8	186.9	24.00	0.115
Isobutyric acid (μg/mg)	52.7	12.3	11.4	11.26	0.249
Pentanoic acid (μg/mg)	207.9	332.4	281.2	23.44	0.078
2-methylbutyric acid (μg/mg)	28.5	5.8	3.0	6.92	0.280
3-methylbutyric acid (μg/mg)	33.8	13.7	8.5	6.13	0.217
4-methylpentanoic acid (μg/mg)	0.2	0.1	0.2	0.05	0.573
Hexanoic acid (μg/mg)	20.3	22.0	9.6	3.55	0.329
Total short-chain fatty acid (μg/mg)	5142.7	6063.4	5740.7	349.1	0.596

[1)]H: supplementation of 20% CP in the early phase (1–14 days) and 18% CP in the late phase (15–28 days); M: supplementation of 18% CP in the early phase and 16% CP in the late phase; L: supplementation of 16% CP in the early phase and 14% CP in the late phase. CP, crude protein.

[2)] mg of cecal digesta.

[3)] Data are expressed in terms of mean (n = 4) values.
