# Supplementary material for: Transcriptome and microbiota analysis reveal differences in the cecum of weaning pigs in response to different dietary crude protein levels
Source: Anim Biosci. 2025 Aug 12;39(1):250135. doi: 10.5713/ab.25.0135 (PMC12754465; doi:10.5713/ab.25.0135)
Supplement: Supplementary file 2 [file ab-25-0135-Supplementary-2.pdf]

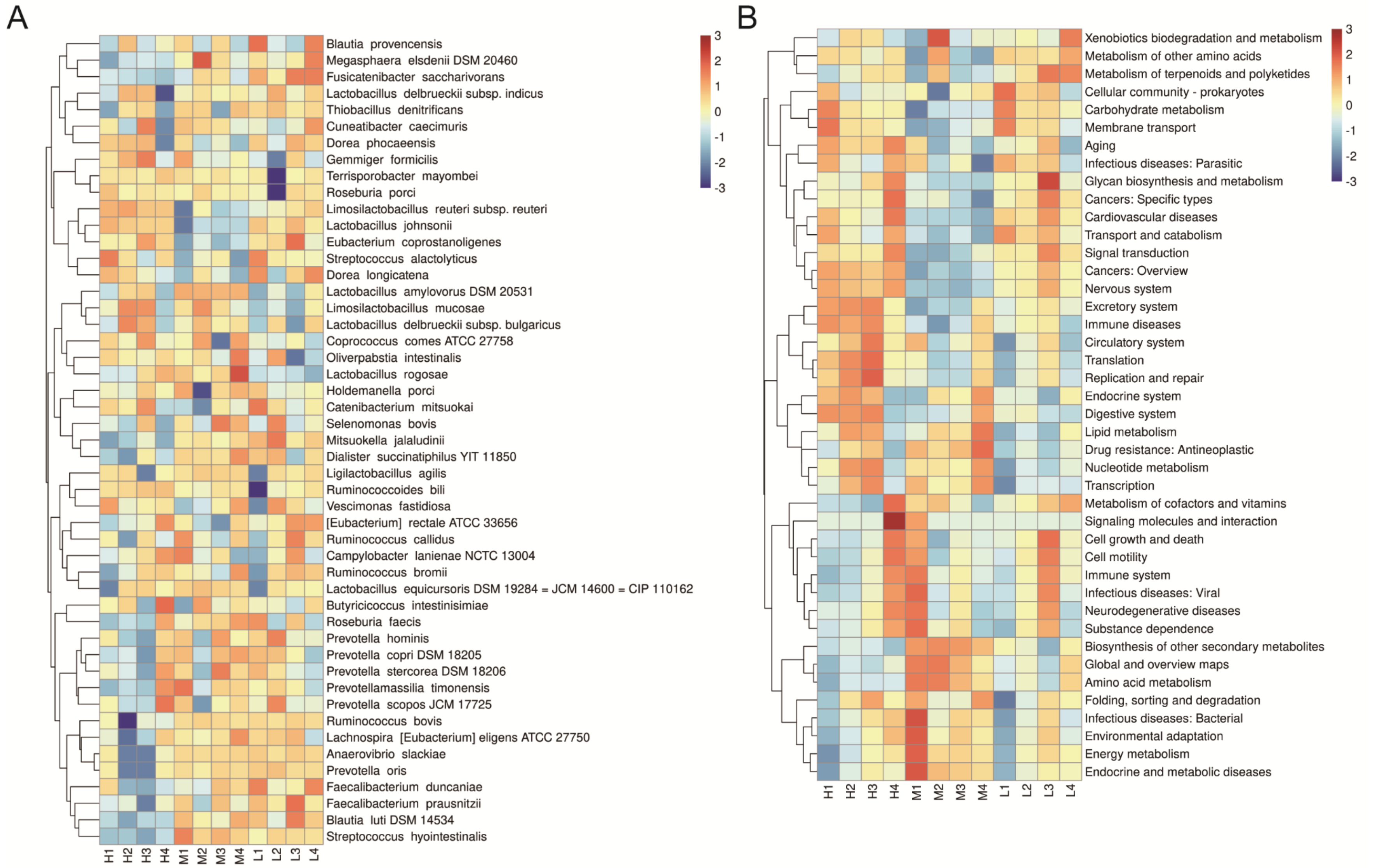

A
Blautia provencensis
Megasphaera elsdenii DSM 20460
Fusicatenibacter saccharivorans
Lactobacillus delbrueckii subsp. indicus
Thiobacillus denitrificans
Cuneatibacter caecimuris
Dorea phocaeensis
Gemmiger formicilis
Terrisporobacter mayombei
Roseburia porci
Limosilactobacillus reuteri subsp. reuteri
Lactobacillus johnsonii
Eubacterium coprostanoligenes
Streptococcus alactolyticus
Dorea longicatena
Lactobacillus amylovorus DSM 20531
Limosilactobacillus mucosae
Lactobacillus delbrueckii subsp. bulgaricus
Coprococcus comes ATCC 27758
Oliverpabstia intestinalis
Lactobacillus rogosae
Holdemanella porci
Catenibacterium mitsuokai
Selenomonas bovis
Mitsuokella jalaludinii
Dialister succinatiphilus YIT 11850
Ligilactobacillus agilis
Ruminococcoides bili
Vescimonas fastidiosa
[Eubacterium] rectale ATCC 33656
Ruminococcus callidus
Campylobacter lanienae NCTC 13004
Ruminococcus bromii
Lactobacillus equicursoris DSM 19284 = JCM 14600 = CIP 110162
Butyricicoccus intestinisimiae
Roseburia faecis
Prevotella hominis
Prevotella copri DSM 18205
Prevotella stercorea DSM 18206
Prevotellamassilia timonensis
Prevotella scopos JCM 17725
Ruminococcus bovis
Lachnospira [Eubacterium] eligens ATCC 27750
Anaerovibrio slackiae
Prevotella oris
Faecalibacterium duncaniae
Faecalibacterium prausnitzii
Blautia luti DSM 14534
Streptococcus hyointestinalis
H1 H2 H3 H4 M1 M2 M3 M4 L1 L2 L3 L4
3 2 1 0 -1 -2 -3
B
Xenobiotics biodegradation and metabolism
Metabolism of other amino acids
Metabolism of terpenoids and polyketides
Cellular community - prokaryotes
Carbohydrate metabolism
Membrane transport
Aging
Infectious diseases: Parasitic
Glycan biosynthesis and metabolism
Cancers: Specific types
Cardiovascular diseases
Transport and catabolism
Signal transduction
Cancers: Overview
Nervous system
Excretory system
Immune diseases
Circulatory system
Translation
Replication and repair
Endocrine system
Digestive system
Lipid metabolism
Drug resistance: Antineoplastic
Nucleotide metabolism
Transcription
Metabolism of cofactors and vitamins
Signaling molecules and interaction
Cell growth and death
Cell motility
Immune system
Infectious diseases: Viral
Neurodegenerative diseases
Substance dependence
Biosynthesis of other secondary metabolites
Global and overview maps
Amino acid metabolism
Folding, sorting and degradation
Infectious diseases: Bacterial
Environmental adaptation
Energy metabolism
Endocrine and metabolic diseases
H1 H2 H3 H4 M1 M2 M3 M4 L1 L2 L3 L4
3 2 1 0 -1 -2 -3

Supplement 2. Heatmap for bacterial distribution and microbial function. (A) Heatmap for the abundance of microbial species in cecal digesta. Distribution of the abundance of 50 dominant species (y-axis) across all samples (x-axis) is shown. (B) Heatmap for the abundance of microbial functions—based on Tax4Fun2 functional categories—in the cecal digesta. Distribution of the abundance of all microbial functions (y-axis) across all samples (x-axis) is shown. H: supplementation of 20% CP in the early phase (1–14 days) and 18% CP in the late phase (15–28 days), M: supplementation of 18% CP in the early phase and 16% CP in the late phase, L: supplementation of 16% CP in the early phase and 14% CP in the late phase. Data were analyzed from 4 replicate pens per treatment. CP, crude protein.
